# Discussions of the Mississippi Valley Dental Association

**Published:** 1878-05

**Authors:** 


### Proceedings of Societies.

### DISCUSSIONS OF THE MISSISSIPPI VALLEY DENTAL ASSOCIATION.

*Continued from Page 170.*

SECOND DAY.—AFTERNOON SESSION.

This session was devoted to the subject, "Artificial Dentures." By permission of the society, Dr. Smith, of Illinois, presented the claims of celluloid as it is now being manufactured and put upon the market.

Dr. Van Antwerp thought celluloid a very good thing. It is an excellent base and I have no use at all for rubber. He had no more failures with it than he formerly had with rubber, and dentures put in four years ago are still good. He thought the dry heat preferable to anything else, and as to the warping of celluloid he thought that could all be overcome. He had used gum teeth for his work almost altogether. He thought absorption under any kind of vegetable plate much greater than under gold. He thought celluloid, however, for the price they have to make work, the best thing that can be had.

Dr. Osmond—There is one thing about rubber in which it has the advantage over every thing else, and that is that you can always warm up the center of the plate and restore the alveolar border. I want to know whether that can be done with celluloid.

Dr. H. A. Smith thought the increased absorption spoken of by Prof. Van Antwerp was due to the closer fit of the rubber plate, than it was possible to get with a metallic plate.

Dr. Hall had used celluloid for over three years, and had found some cases that with all the care and skill he possessed he was not able to treat so successfully as with rubber. Had found discoloration a serious difficulty; had also found it exceedingly difficult to repair if you have to unite new material with old. Did not think it would stand the constant use that rubber would. In the course of a year or two it will yield, and in cases where old ladies smoked, they did not last six months, but just literally burned up.

Dr. McMillan said he had three partial plates of celluloid, the first that came out, and they had been worn seven years without change.

Dr. Meridith—About ten or twelve years ago I put in a rubber plate for a patient, which gave out sometime since, and he went to Louisville and had a celluloid plate put in and now he complains of it. He says the camphor taste is objectionable, and that the fit is not so perfect as with the rubber plate.

Dr. Hall had three cases where patients complained of the camphor taste, and he had replaced the plates with rubber, since which time they had made no complaints.

Dr. Cameron—I commenced on rose pearl in 1868, and have used celluloid ever since. Have never made a rubber plate since. I have never noticed any difficulty of the nature Dr. Hall speaks of. I have made a great many celluloid plates for patients who had been using rubber, and whose mouths were in such bad condition that I would not take the impression until they had gone without their teeth for a week or two, and the difficulty did not appear again under the use of celluloid. I have had no trouble with the working of the celluloid; I have occasionally failed as I have with everything else, but I did not always blame the material. As to the discoloration, persons who chew and smoke will discolor any plate, even gold. I am decidedly in favor of celluloid, and opposed to rubber. Last year an old practitioner said in this room, that he had never seen a mouth that he thought was effected by rubber. Now while I believe that gentleman told what he thought was true, I can, with equal sincerity say, that I have never seen a mouth that I thought was not effected by it. I speak of full sets.

Dr. Jameson—I have had little or no experience with celluloid, but I have had the same difficulty with reference to rubber plates that Dr. Cameron speaks of. Of the mouths that I have examined, I have not found three that were not more or less affected by the use of rubber plates; some of them very seriously, others not so bad. I believe a gold plate with a rubber or celluloid attachment is the best plate, but I believe that in ninety-nine out of one hundred cases a silver plate is preferable to a rubber or any vegetable material, and I would use it with rubber if it could be done, but it can not as the sulphur in the rubber would destroy the silver.

Dr. H. A. Smith—This difficulty with rubber plates is either because of the close fit or the poisonous substance in the rubber. Dr. Judd says positively it is poisonous; Dr. Atkinson says it is not.

Dr. Abraham Roberts, of Mass., cited some cases where patients had suffered from the use of red rubber, and been entirely relieved by the substitution of black rubber.

Dr. Leslie had never known an instance where black rubber had been substituted for vermillion that any difficulty had been experienced afterwards. Did not think the close fit would cause the difficulty, but thought if the plate was not removed over night that it might. Subject passed.

### EVENING SESSION.

The society having assembled at 7:30 the fifth subject, "Operative Dentistry," was taken up and discussed as follows:

Dr. Taft—This is a subject fraught with some interest now in a new aspect. It is claimed away off East from whence all the light comes that a new method pertaining to operative dentistry has been discovered, and is now put in practice and presented to the profession. Now what is that startling discovery? It is just this, that gold, as a filling for teeth, is totally discarded. Gutta percha, Hill's stopping, and I don't know but possibly mud, are the better things for the purpose. It has been claimed that gutta percha fillings will wear twenty years. Now our experience is, that in many cases it will wear out in a few months, or in two or three years at the most. In the reported proceedings of the Odontological Society of New York, Dr. Flagg said that gold as a filling for teeth was entirely discarded. This new departure has advocates in various parts. Dr. Palmer, of Syracuse, N. Y., has been experimenting and the drift of his argument would lead us to conclude that gold is not a good thing to fill teeth with, and that these plastic materials are. Dr. Flagg, of Philadelphia, proclaims the same thing. If what Dr. Chase, of St. Louis, says of the incompatibility between gold and tooth substance is true, and the fact that stannous or tin gold is entirely compatible—we must conclude that gold is not a good substance for filling teeth. Now those of us who have filled teeth from twenty to thirty years with gold, and find them to maintain their integrity right along ever since, wonder how it is that gold is so entirely unfit for filling teeth. If something better than gold and a great deal less expensive has been found for filling teeth, it will be a most happy thing, for gold costs money, and it is sometimes difficult to get just what we want in that line, but I never heard of any difficulty in getting gutta percha, which is always good (?) or the stannous gold from St Louis, which is always good. (?)

Dr. Keely—I rise to defend Dr. Flagg against the attack of Dr. Taft. A few years ago I remember Dr. Flagg was here and was then the champion of Morgan's plastic gold, and he filled a tooth with it in three minutes, and I told him I was sorry I was so behind the times, that I had two or three students in my office who were pretty good operators, but I didn't think that all of us together could fill a tooth in three minutes. According to his idea, if you met a friend on the street you could say to him, open your mouth, sir, and whack in the filling before he had time to close it. Now Flagg is an adept at filling teeth—with amalgm, etc. The only objections I have to Flagg is, that up at Niagara one year he was talking about that, and I was so taken with it that I engaged a ton, and told him to send it C. O. D., and he never sent it.

Dr. C. S. Smith, of Ill.—Some years ago I was a student in a dental college where a certain professor discovered a new method for bleaching teeth, which was a process for generating and applying chlorine gas. As I was young and unexperienced, I armed myself with all the apparatus for bleaching teeth, and started West. Before going West, however, I went to New York, where I passed several days at the office of Dr. Atkinson. Of course I dilated to a considerable extent upon the remarkable qualities of my wonderful tooth bleaching process. While I was there, a certain notable dentist came in to have some operations performed upon his teeth by Dr. Atkinson. One was an eye tooth which needed bleaching, and I was called upon to bring my gasometor into requisition, and I did so. As a result the patient was very nearly stiangled, but Dr. Atkinson by the exercise of his well known will power, kept him from committing violence. I didn't have the pleasure of meeting the patient, till several years afterward when I saw him at Sarotoga, and that eyetooth was about the color of my coat sleeve. At a meeting of the Odontological Society which I had the pleasure of attending some time ago, Dr. Bogue was performing some experiments with various bleaching compounds, and at that time he stated that there were some substances that would have a directly opposite effect, and mentioned chlorine gas as one of them.

Dr. Osmond said that Dr. Fletcher, of England, had stated that comparatively few plugs put in in this country would stand the ink test, and he wanted to know whether Dr. Fletcher had performed his experiments alone, or in the presence of some one else. Of course nothing could get around Dr. Fletcher's amalgam. (?) He had obtained some of his filling, and found it nothing but oxy-chloride.

Dr. H. A. Smith thought there might be something in the new departure which had been so ably ridiculed. He thought Dr. Taft did wrong to class Dr. Palmer, of Syracuse, with Dr. Flagg and that class of men. His electrical theory is, of course, but a theory needing to be proved, but he thought Dr. Palmer a sincere investigator. Some kind of plastic filling he thought was necessary for cavities of such a nature or in such a position that gold could not successfully be used. There is too much claimed for filling teeth. They will decay, and dentists ought not to promise that they will not. He thought it would be an outrage upon the resources of nature, to suppose that a good plastic filling would never be discovered; but he had never found any amalgam yet that was reliable, whereas gold is just what you make it.

Dr. Hunter wanted to object to the new coined word dentos—they meant dentine. He thought there was a demand for some plastic material, but as yet there was nothing of that nature that was reliable; however he expected to see the time when there would be a plastic filling that would supersede gold. Those who discard amalgam altogether make a great mistake.

Dr. Keely—The best operators in the land make mistakes by filling teeth with gold; especially in the case of children. It is not only a very severe operation to which to subject a child, but we also run the risk of injuring the teeth. I believe, as firmly as I believe that gold is the best filling under most circumstances, that tin is the best filling under these circumstances. You can put it in place more readily, in less time and by the use of less force, and it will preserve the tooth better than gold. There is something about tin that is very congenial to the senitive dentine of young children. You might as well bid farewell to the tooth at once as to put gold into the mouth of a young child. Amalgam has its place, but it should be used seldom. I think with Dr. Hunter that the time will come when a plastic material will take the place of gold. I saw a gutta percha filling the other day that had been in twelve years; also an oxy-chloride filling that had been in the same length of time.

Dr. Osmond spoke of a tooth of Mr. Anderson, in which a gold filling had remained for fifty-five years It was in the posterior proximate surface of the right superior canine. He thought this was some argument in favor of the durability of gold.

Dr. Taft said that fillings often failed at the necks of teeth which was not always because of an inefficient operation. In order to make a good filling at the cervical wall the cavity must be properly formed. He then described by means of diagrams on the board how the cavity should be prepared, and the fillings inserted. He also thought that gold was not always the best thing for filling teeth. Its conduction of heat is a very great objection to it, and that is one reason why tin is sometimes better than gold.

Dr. Canine said he had heard Dr. Flagg's statement with considerable interest, and while he could not endorse his views in full, he thought there was some truth in them, particularly with some people and some operators. He cited the case of a lady who had had several teeth filled, all with gold except one which was filled with gutta percha, because the nerve in that tooth was absolutely bare. Seven years afterward the lady came back to the dentist and all the gold fillings were gone, while the gutta percha filling remained almost perfect. He thought that the average dentist saved more teeth by the use of amalgam fillings than they did with gold fillings.

Dr. Berry said he knew a man who had twelve gold fillings that had been in forty-two years and were still good.

Dr. Meridith thought the best material for filling teeth was the osteo-plastic filling in the various forms. It is a non-conductor of heat and cold, allays sensitiveness and forms an absolute attachment to the walls, which no other material does, so that it forms as nearly a perfect tooth as is possible to conceive of. It only lacks one thing and that is durability; it will wash out. Gold stands second in the list. I never saw decay start around white fillings and I don't believe any other man ever saw it while it was perfect. Of course after it was washed out and the dentine was exposed it would decay.

FRIDAY MORNING SESSION.

The society having assembled at 9:30 a. m., discussion on operative dentistry was continued as follows:

Dr. Sage—I want to add my testimony to the remarks of Dr. Osmond as to the ink test. Some years ago a lady came into my office at Sandusky where I was practicing at the time, to have some teeth extracted, but I prevailed upon her to have them filled, and I did fill three of them. She was taken sick and did not come back to my office for two or three months, and when she did return I was absent and my partner concluded to extract the teeth including the three which I had filled. A few days afterward Dr. E. J. Waye came into my office with a letter of Dr. Fletcher's, which stated that a filling of cohesive foil could not be perfectly tight. I gave him one of the teeth which had not been out of the mouth more than a day, and was subjected to the ink test and did not leak in the least. Also cited some cases where tin fillings had been more durable than gold.

Dr. Taft thought one great advantage in the use of tin was the fact that it is a non-conductor of heat. Dr. Watt had said that it was antiseptic in its character, but he thought the great benefit was in the fact that it was a non-conductor.

Dr. Leslie said he wished to express his indignation at the feeling which seemed to be gaining ground, that amalgam was better than gold for a filling. He had a tooth in his own mouth which had been filled with gold for thirty years and was still good. But since the introduction of cohesive foil, many operations had been performed which would not have been performed many years ago; and from this fact many people had been led to believe that filling was not so well done now as formerly. He also thought the instruments formerly used were better than the instruments of to-day for the purpose for which they were intended; and they had smooth points and much larger points than they have to-day. If he wanted a man to spoil a gold filling he could not conceive of a better method than to break up the cohesiveness of the gold by the use of the small pointed instruments of to-day. He said that by his process of manufacturing crystal gold he felt confident that he had obtained the very best possible filling for teeth. That he was not pushing it for the purpose of making money, and was perfectly willing to wait until its own merits recommended it to the use of the profession.

Dr. Osmond said he had had some experience with crystal gold, and had failed in every instance.

Dr. Hunter said that he wanted to make an explanation. It had been reported that he had declared in favor of the new departure, whereas he had only meant to say that those men who confined themselves exclusively to gold filling made a mistake, and that a good amalgam filling was better than a poor gold one.

Dr. Taft—I don't know of any men who use gold exclusively.

Dr. Hunter—I mean those who don't use amalgam at all.

By motion of Dr. Taft, the sixth and seventh subjects were passed, and the eighth subject, Suppuration of the Gums, and its Treatment, was taken up for discussion.

Dr. Jay referred to some of the causes of suppuration. The first and most frequent cause was pure neglect of cleansing the mouth properly, which caused chemical decomposition, and inflammatory action. The accumulation of salivary calculus will sometimes produce it. As to the treatment, in the first place it is necessary to cleanse the gums properly. He usually used some stimulating wash; tincture of myrrh being a favorite remedy. He cited the case of a gentleman who came to him with his mouth in a terrible condition, and he asked him how long it had been since he washed his mouth. The man said he had never washed it in his life. He was a tobacco chewer, and between his teeth were wedged pieces of tobacco which had remained there until the gums were partially absorbed and very highly inflamed. He had told him to go to the drug store and get a soft tooth brush and go home, get some white oak bark, and make an ooze of it, and wash his mouth with it, and after he had used it for some time, to return and report to him. As usual in such cases, the man went back on him, and never reported afterward.

Dr. Cassidy said that he did not see why the use ot tobacco should injure the gums unless it were fine cut tobacco, which might injure them by the quantity of saccharine matter which it contained. He had seen gums injured by that. For suppuration of the gums he had prescribed tannin dissolved in glycerine with good effect.

Dr. Rawles said that ordinary suppuration of the gums was easily cared for by local treatment. But when it was to be referred to a mercurial taint, he did not think it possible to obtain definite and permanent relief by local treatment alone. In such cases systemic treatment must also be resorted to. He thought this subject too important to be properly discussed in the limited time which yet remained, and he suggested to the executive committee that they put this subject on the next programme.

Dr. Taft—I think that the system sometimes becomes involved from the local difficulty, which, when removed, the whole matter will be remedied. When the systemic condition depends upon the local difficulty, then it is possible to so change these local conditions by local treatment, independent of any systemic treatment, that the symptoms will be relieved. Many times the systemic treatment would amount to nothing, while the local condition remains. The poison in the system may be somewhat neutralized by the proper agent, but it will not amount to anything if the supply is kept up all the time. The supply must be cut off.

Dr. Meridith thought the great majority of cases of suppuration of gums might be cured by local treatment. He had been remarkably successful in several cases, after the removal of all foreign matter, by packing a little piece of cotton very delicately around the fibrous part of the gum to allow the suppuration to exude properly.

